# Kaposi’s sarcoma-associated herpesvirus terminal repeat regulates inducible lytic gene promoters

**DOI:** 10.1128/jvi.01386-23

**Published:** 2024-01-19

**Authors:** Yoshihiro Izumiya, Adhraa Algalil, Jonna M. Espera, Hiroki Miura, Chie Izumiya, Tomoki Inagaki, Ashish Kumar

**Affiliations:** 1 Department of Dermatology, School of Medicine, University of California Davis, Sacramento, California, USA; 2 Department of Biochemistry and Molecular Medicine, School of Medicine, University of California Davis, Sacramento, California, USA; 3 Midwestern University College of Dental Medicine, Glendale, Arizona, USA; Lerner Research Institute, Cleveland Clinic, Cleveland, Ohio, USA

**Keywords:** Kaposi's sarcoma-associated herpesvirus, transcriptional regulation, reactivation, RNA polymerases, latency, enhancer, BRD4, CHD4, herpesviruses

## Abstract

The Kaposi’s sarcoma-associated herpesvirus (KSHV) genome consists of an approximately 140-kb unique coding region flanked by 30–40 copies of a 0.8-kb terminal repeat (TR) sequence. A gene enhancer recruits transcription-related enzymes by having arrays of transcription factor binding sites. Here, we show that KSHV TR possesses transcription regulatory function with latency-associated nuclear antigen (LANA). Cleavage under targets and release using nuclease demonstrated that TR fragments were occupied by LANA-interacting histone-modifying enzymes in naturally infected cells. The TR was enriched with histone H3K27 acetylation (H3K27Ac) and H3K4 tri-methylation (H3K4me3) modifications and also expressed nascent RNAs. The sites of H3K27Ac and H3K4me3 modifications were also conserved in the KSHV unique region among naturally infected primary effusion lymphoma cells. KSHV origin of lytic replication (Ori-Lyt) showed similar protein and histone modification occupancies with that of TR. In the Ori-Lyt region, the LANA and LANA-interacting proteins colocalized with an H3K27Ac-modified nucleosome along with paused RNA polymerase II. The KSHV transactivator KSHV replication and transcription activator (K-Rta) recruitment sites franked the LANA-bound nucleosome, and reactivation evicted the LANA-bound nucleosome. Including TR fragments in reporter plasmid enhanced inducible viral gene promoter activities independent of the orientations. In the presence of TR in reporter plasmids, K-Rta transactivation was drastically increased, while LANA acquired the promoter repression function. KSHV TR, therefore, functions as an enhancer for KSHV inducible genes. However, in contrast to cellular enhancers bound by multiple transcription factors, perhaps the KSHV enhancer is predominantly regulated by the LANA nuclear body.

**IMPORTANCE:**

Enhancers are a crucial regulator of differential gene expression programs. Enhancers are the cis-regulatory sequences determining target genes’ spatiotemporal and quantitative expression. Here, we show that Kaposi’s sarcoma-associated herpesvirus (KSHV) terminal repeats fulfill the enhancer definition for KSHV inducible gene promoters. The KSHV enhancer is occupied by latency-associated nuclear antigen (LANA) and its interacting proteins, such as CHD4. Neighboring terminal repeat (TR) fragments to lytic gene promoters drastically enhanced KSHV replication and transcription activator and LANA transcription regulatory functions. This study, thus, proposes a new latency–lytic switch model in which TR accessibility to the KSHV gene promoters regulates viral inducible gene expression.

## INTRODUCTION

There is growing awareness of the need to understand the spatial and temporal organization of the genome structure and its role in regulating gene expression. Given that nuclear enzymes and transcriptional factors are in limited supply and cannot be everywhere in the nucleus at the same time, the nuclear architecture (including chromatin structure and protein distribution) must allow for these molecules to be concentrated at the proper time and place for coordinated gene expression to occur. Understanding the three-dimensional (3D) structure of the genome and its impact on neighboring genomic elements is, therefore, critical to understanding gene regulation (1–5). The significance is also highlighted by the fact that the deregulation of genomic interactions by mutations of intragenic genomic regions and nuclear remodeling factors such as SWItch/Sucrose Non-Fermentable (SWI/SNF) frequently leads to diseases (6, 7).

Assisted by the three-dimensional genomic architecture, gene enhancers are known to act in *cis* and an orientation-independent fashion by forming genomic loops to increase frequencies for transcription initiation at promoters. By forming genomic looping with multiple promoters, the enhancer DNA fragment neighbors and activates many promoters at the same time (8, 9). A larger genomic region collectively bound by an array of transcription factors at higher density, hence harboring a higher density of transcription enzymes, is called a super-enhancer (10). Cellular proteins, such as mediator complex subunit 1 (MED1) and bromodomain-containing protein 4 (BRD4), are known to be located at the super-enhancer region, and those proteins further compartmentalize nuclear condensates with their intrinsically disordered domain for maintaining selective gene expression, hence cell identity (11). The genomic interaction between enhancer and promoter mediated by transcription-related enzymes positions the RNA polymerase II (RNAPII) pre-initiation complex at specific genomic sites for sensitizing inducible gene expressions with specific signaling events that activate enhancers (2, 11–16).

The Kaposi’s sarcoma-associated herpesvirus (KSHV) viral genome consists of an approximately 140-kb unique coding region flanked by multiple terminal repeats (TRs) of 801 bp with high GC content (17). KSHV genomes persist in latently infected cells as episomes via tethering to the host cell chromosomes. During latency, the expression of lytic viral genes is poised to be transcribed, and only a few latent genes are actively expressed and translated (18). Among these latent genes, ORF73 encodes latency-associated nuclear antigen (LANA), which plays a crucial role in latent episomal DNA replication and segregation during host cell mitosis. The TR contains a DNA replication origin called ori-P that consists of two LANA-binding sites (LBSs): a higher affinity site (LBS1) and a lower affinity site (LBS2) followed by an adjacent 32-bp GC-rich segment (18). Episome maintenance requires at least two LBS1/2 binding sites, and the viral genome consists of 30–40 TRs (17, 19). A crystal structure demonstrated that the LANA DNA-binding domain (LANA_DBD_) mainly exists as a dimer in solution. Five LANA_DBD_ dimers can interact end-to-end to form a decameric ring with an exterior diameter of 110 Å and an interior diameter of 50 Å, and the inner diameter is sufficiently large to accommodate double-stranded DNA (20). DNA binding further induces oligomerization of LANA_DBD_, and a hydrophobic interface between LANA dimer to form the decameric ring is essential for cooperative DNA binding and DNA replication, hence episome maintenance (20). The specific LANA’s TR DNA binding also increases up to 600–800 LANA copies [2 (dimer) × 5 (decametric ring) × 2 (two binding sites per TR unit) × 30–40 (TR copies per episome)] to locate a single KSHV episome. As a result, LANA can be seen as LANA dots in KSHV-infected cells with immunostaining (21). Because KSHV latent chromatin is circular (22–25), the TRs always localize in relatively close proximity to the unique region that encodes all of the inducible genes. Those inducible promoters are known to be activated by KSHV replication and transcription activator (K-Rta) protein. Isolated reporter constructs showed that at least 33 lytic gene promoters can be activated by K-Rta in 293 cells (26). Interestingly, previous studies indicated that TR possesses transcription regulatory functions in reporter assays (27, 28).

In this report, we show that the TR is (i) encoding an array of transcription factor (LANA)-binding sites, (ii) recruited by transcription-related enzymes including BRD4, (iii) modified by a histone H3K27Ac mark, (iv) expressing nascent RNAs, and (v) possessing an orientation-independent transcription activation function. We propose a model in which KSHV TR is a large transcription regulatory domain (enhancer) for KSHV inducible gene promoters (unique region).

## RESULTS

### Latent KSHV chromatin modification maps

To understand the KSHV latency–lytic switch, we first revisited a comprehensive histone modification of the KSHV episomes in naturally infected cells. We used a public database and our cleavage under targets and release using nuclease (CUT&RUN) data sets. If having a specific regulatory genomic domain is necessary for maintaining inducible KSHV latent chromatins, cell lines that can produce infection virions with stimulation should conserve similar genomic domains in KSHV latent chromatin. Four histone modifications primarily associated with active transcription (H3K4me1, H3K4me2, H3K4me3, and H3K27Ac) and repressive marks (H3K27me3, H3K9me3) were examined. While we tried to locate H3K9me3, we could not identify reasonable peaks with two independent antibodies.

**Fig 1:**
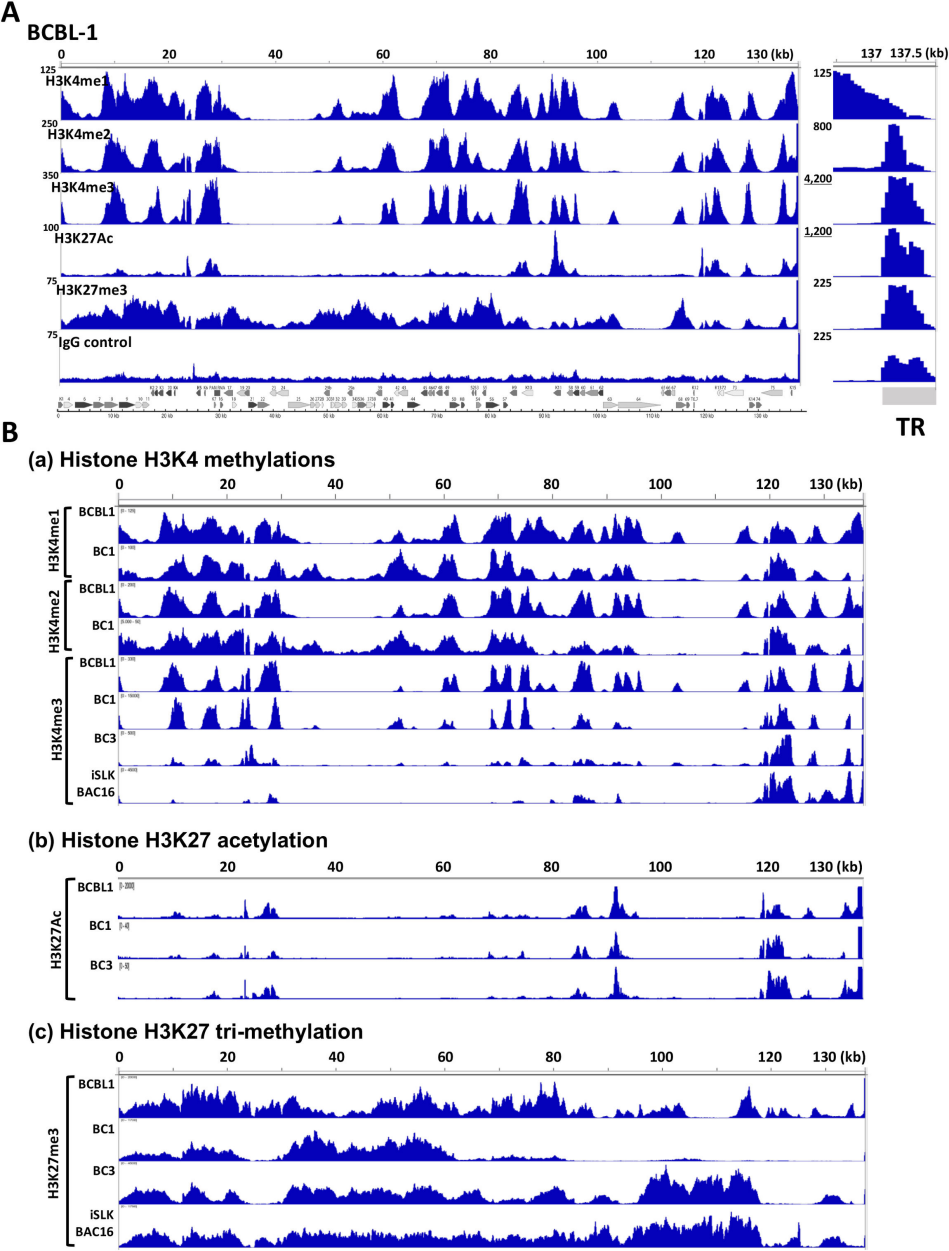
KSHV chromatin modifications. (**A**) KSHV latent chromatin modifications in BCBL-1. The position of indicated histone modification was examined with CUT&RUN analyses. The respective peak heights after CPM normalization are indicated on the left panel. The KSHV ORF map was depicted at the bottom of the panel. The terminal repeat region is zoomed at the right after adjusting the scale of peak ranges. Sequence enrichments seen more than 10 times compared with that of the unique region were underlined. (**B**) Differential chromatin modifications in KSHV-infected cells. Selected histone modifications were compared among KSHV-infected cells. The cell line name was depicted on the left of the panel. The sequence reads (without CPM normalization) were plotted on the KSHV genome. The H3K27Ac ChIP-seq data for BC1 and BC3 are adapted from SRR9956027 and SRR9956035, respectively.

Figure 1A depicts histone modifications of KSHV latent chromatin in BCBL-1 cells. The count per million (CPM) normalized peaks were mapped on KSHV genomes. The results showed that four active histone marks are clustered mainly at genomic loci encoding early and immediate-early genes consistent with previous reports (29, 30). The H3K27Ac marks were restricted at two Ori-Lyt regions, PAN promoter, viral interferon regulatory coding loci, LANA promoter, ORF75 promoter, and K15 promoter regions (Fig. 1A). Although H3K4me1 and H3K4me2 marks are colocalized with H3K4me3 marks, H3K4me1 and H3K4me2 modifications were more broadly distributed around the H3K4me3 marks. The H3K4me3 modification showed sharper peaks than H3K4me1 and me2 marks in BCBL-1 (Fig. 1A). While it is difficult to compare between peaks at the unique genomic region of the KSHV genome (one copy per genome) and that of TR fragments (30–40 copies per genome), there are still noticeably strong H3K4me3 and H3K27Ac signals (more than 10 times) at the TR regions but not with the H3K4me1, 2, or H3K27me3 (Fig. 1A, right panel). On the other hand, repressive marks (H3K27me3) were localized more broadly in BCBL-1. Other KSHV naturally infected primary effusion lymphoma cells or experimentally infected iSLK cells showed similar histone modification occupancies for active histone marks, especially for H3K4me3 and H3K27Ac marks. However, H3K27me3 had different degrees of signals at late gene cluster regions. With that, BC3 cells showed more H3K27me3 modification at late gene cluster regions, while BC1 cells showed little signals at the same regions (Fig. 1B). The experimentally infected iSLK cells also showed patterns more resembling BC3 and largely occupied by H3K27me3-modified histones. These results indicate that maintaining active genomic regions is a more conserved trait than the repressive mark for KSHV latent chromatin.

### TR region is actively transcribed in latently infected cells

**Fig 2:**
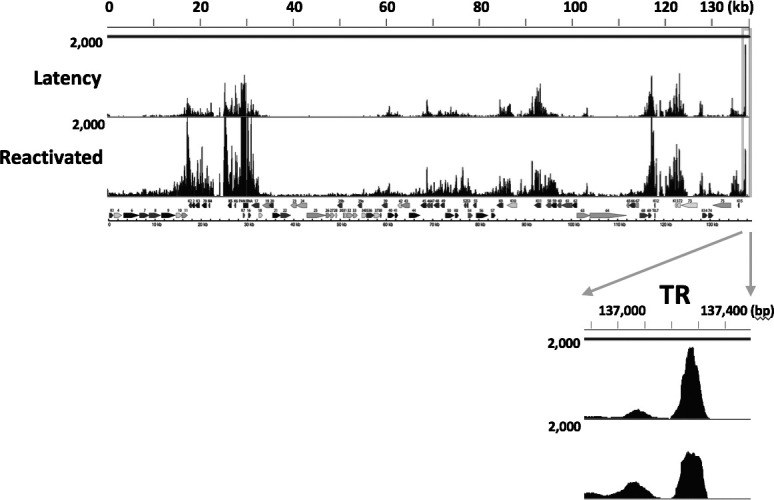
TR region expresses nascent RNAs. GRO-seq signals aligned to the KSHV genome during latency and upon reactivation. GRO-seq data were obtained from the public data (32). Sequence reads were mapped to the KSHV genome. The KSHV ORF map was depicted, and the terminal repeat region is zoomed in at the bottom of the panel.

Significant modifications by the active histone marks (H3K27Ac and H3K4me3) at TR suggested that TR may possess an enhancer function. To assess if TR is an active enhancer, we next examined the transcriptional activity at TR. Nascent RNA sequencing, which measures enhancer RNAs, provides more direct evidence of enhancer activity. We, thus, reanalyzed the previous GRO-sequence data to identify nascent transcribing RNAs in the TR region and mapped nascent RNAs on the KSHV genome (31). The results showed that TR is actively transcribed during latent infection, and reactivation slightly reduced the amount of nascent RNAs from TR (Fig. 2).

### Protein interaction among K-Rta and LANA-interacting proteins

To gain insights into the latency–lytic switch mechanism, we next focused on the protein–protein association networks of KSHV LANA and K-Rta-interacting transcription-related enzymes by using the STRING database (33). In this analysis, functional interactions between two proteins were visualized by the number of lines among protein nodes. The line between two nodes indicates either experimentally validated interaction, coprecipitated proteins in the proteomics database, or predicted interaction based on coexpression and functionality (33). An increased number of lines between two nodes usually suggest higher confidence in the protein–protein interaction.

**Fig 3:**
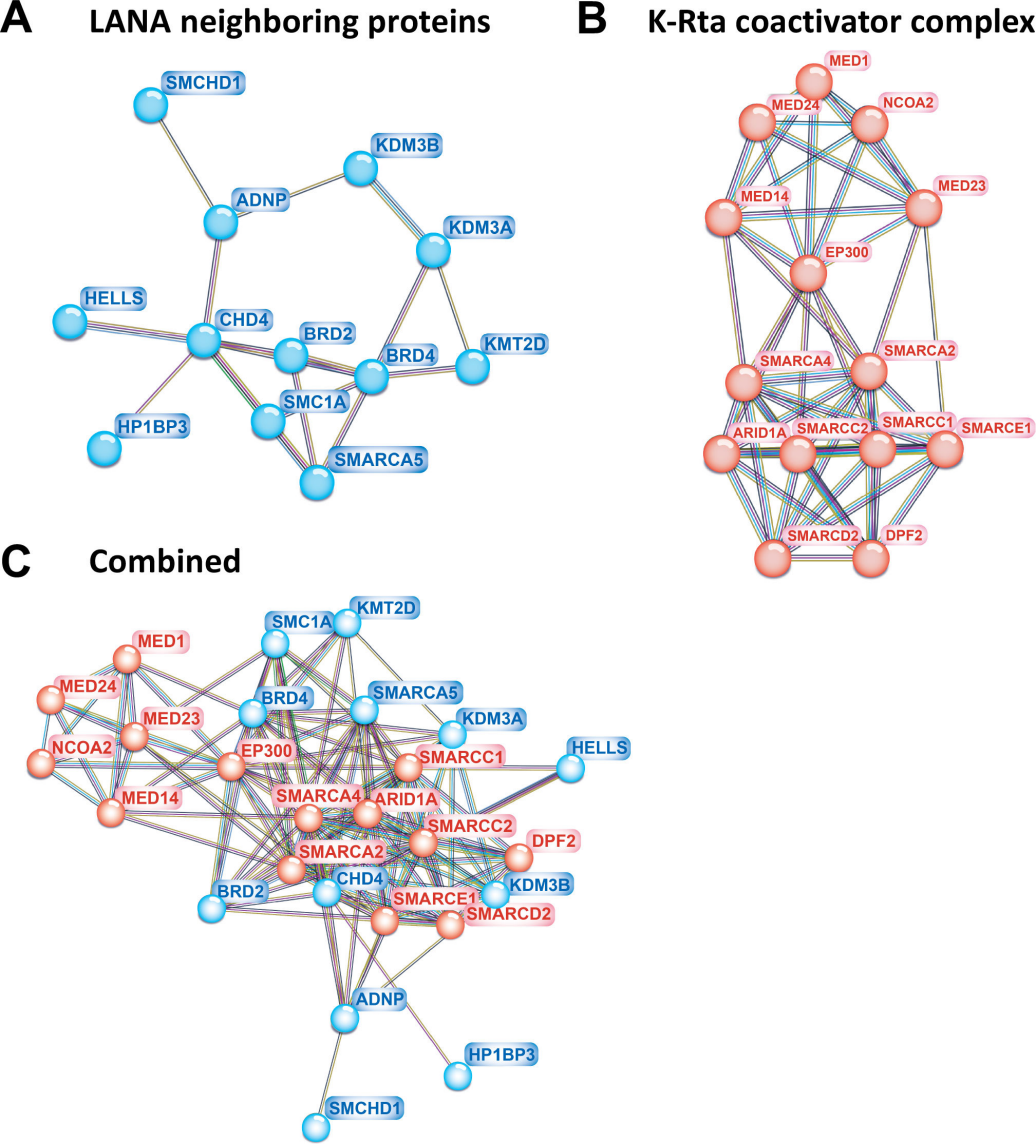
LANA and K-Rta protein complexes. (**A**) LANA neighboring protein in iSLK cells. Previously identified LANA neighboring chromatin binding proteins by proximity biotin labeling with recombinant KSHV were visualized with STRING (33). The line between nodes indicates putative protein interactions based on experimental verification (purple line), curated databases (light blue), text mining (yellow-green), coexpression (black line), and protein homologies with other organisms (light purple). (**B**) K-Rta and RNA polymerase II interacting protein on chromatin. Previously identified K-Rta interacting proteins that are recruited to RNAPII by the RIME study were visualized with STRING. The RIME showed the formation of a putative large protein complex containing SWI/SNF and mediator complex. (**C**) Predicting protein interactions among LANA and K-Rta complexes during reactivation. Theoretical protein complex interactions in the presence of K-Rta are simulated with STRING (33). Proteins indicated by blue ovals are those in close proximity to LANA identified by proximity biotin labeling while proteins indicated by red ovals are those interacting with K-Rta identified by the RIME assay. The number of lines between two proteins represents the overall confidence of the putative protein–protein interactions.

KSHV LANA is essential for establishing and maintaining latent infection, and we previously identified proteins localized in proximity to LANA (24). Among the proteins localized in proximity to LANA (Fig. 3A), we showed that cellular CHD4 protein plays a key role in establishing and maintaining latent infection (24). Proximity-biotin labeling with recombinant KSHV also confirmed that LANA interacts with BRD4 and also identified components of the Imitation SWItch complex and several others as LANA-interacting proteins [Fig. 3, blue oval (24)]. In addition, to reveal how K-Rta triggers KSHV reactivation, we also reported cellular proteins that are induced to interact with RNAPII in the presence of K-Rta during KSHV reactivation. In the latter study, we applied rapid immunoprecipitation mass spectrometry of endogenous protein (RIME) (34). The method is suitable for identifying transcription factor complexes on chromatin. By identifying proteins that interact with RNAPII and K-Rta during reactivation on chromatin, we isolated critical proteins for K-Rta transactivation (34). The study identified that K-Rta interacted with NCoA2, SWI/SNF, and mediator complex, and these proteins may form a large protein complex (Fig. 3B). The results were consistent with previous reports that showed K-Rta interaction with NCoA2 and SWI/SNF and mediators (35, 36). To provide insights into dynamic protein interactions between two protein complexes during reactivation, we combined and visualized putative protein interactions among the two protein complexes (Fig. 3C). The result showed that LANA-interacting proteins (blue ovals) may interact with components of the K-Rta complex (red ovals), when they were recruited to proximity during reactivation. Nearly all, except two (SMCHD1 and HP1BP3) LANA-interacting proteins, were predicted to interact with at least one of the components of the K-Rta complex (Fig. 3A). The protein interaction model suggests that components of the LANA protein complex at TR (during latency) can be flexible when K-Rta brought its protein complex near the LANA binding sites during reactivation.

### Determining LANA-interacting protein recruitment sites on the KSHV genome

**Fig 4:**
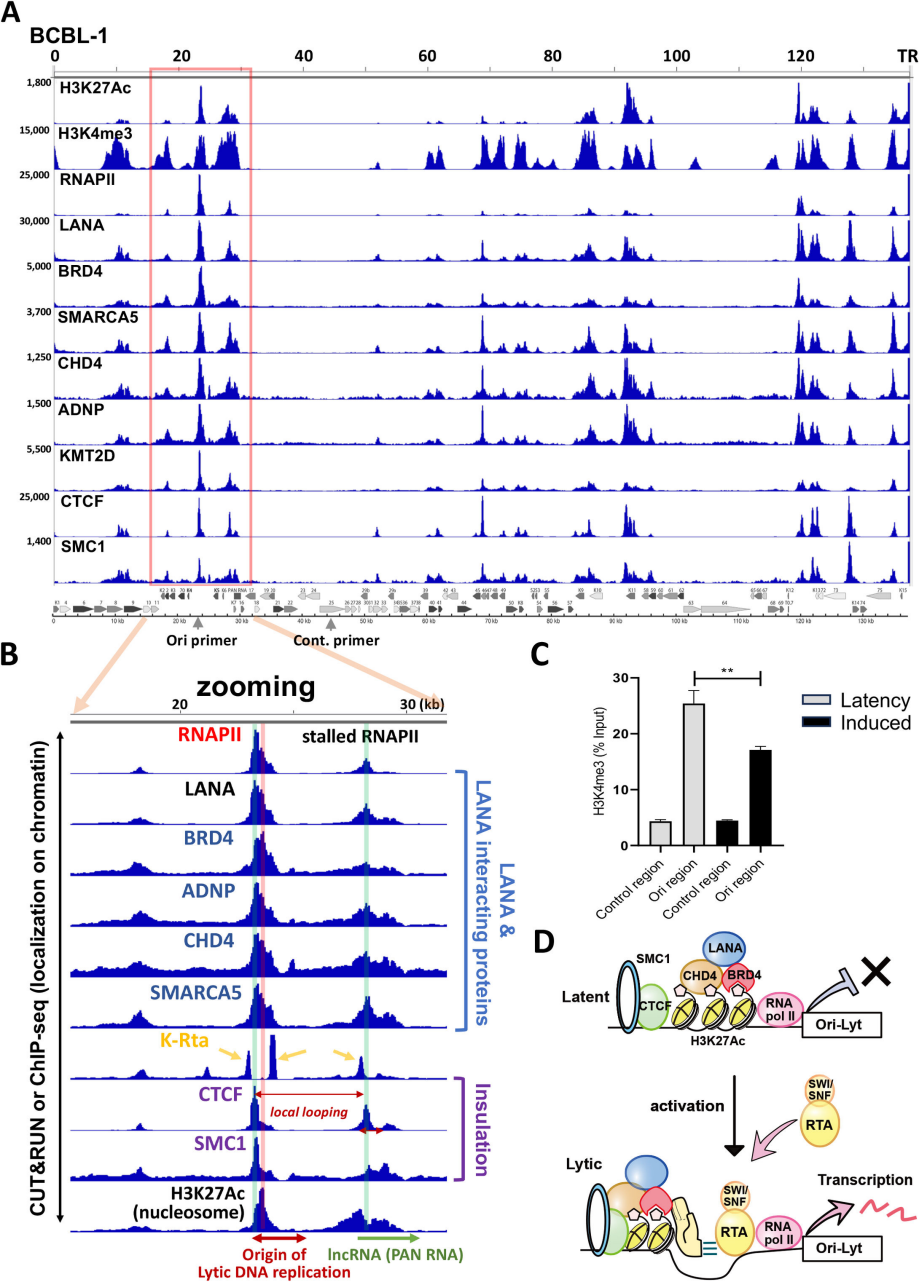
Overview of LANA complex recruitment sites on KSHV genome in BCBL-1. (**A**) CUT&RUN. LANA-interacting protein recruitment sites were determined by CUT&RUN and depicted on the KSHV genome without CPM normalization. The range of sequence reads is shown on the left of the panel. The KSHV ORF map is depicted at the bottom of the panel. The enrichment of sequence reads at TR over the unique region is presented in Table 1 separately. The position of primers used for (**C**) is indicated at the bottom of the panel. The same sets of CUT&RUN were also performed with BC-1 cells and presented in Fig. S1. (**B**) Zooming at Ori-Lyt and PAN RNA promoter in the unique region. One of the LANA complex-recruited sites at the unique region is zoomed, and positional association among CTCF/SMC1 (purple), H3K27Ac nucleosome, LANA complex recruitment site (blue), and K-Rta binding sites (yellow) is emphasized. The position of the H3K27Ac-nucleosome (red shadow) and CTCF binding sites (green shadow) is also indicated. The association with the frequencies of genomic looing is depicted in Fig. S2. The origin of Lyic DNA replication and PAN RNA regions are highlighted at the bottom of the panel. K-Rta ChIP-sequence data are adapted from the public database (37). (**C**) The nucleosome eviction at the origin of DNA replication during reactivation. TREx-K-Rta BCBL-1 cells were left untreated or treated with doxycycline (1 µg/mL) and TPA (20 ng/mL) for 48 h. CUT&RUN was performed using the H3K4me3 antibody. The percent of input was calculated and shown in the panel. The H3K4me3 non-binding region (control region) was used as a negative control. An unpaired *t*-test was used for calculating the *P*-value. ^**^P < 0.05. (**D**) Proposing latency–lytic switch model. Through sequence-specific DNA binding, K-Rta recruits the SWI/SNF complex at K-Rta binding sites. The SWI/SNF complex slides/evicts nucleosomes that the LANA complex is tethering. The LANA complex binds the H3K27Ac through direct histone binding or BRD4. The destabilization of the LANA complex and nucleosome eviction facilitates RNAPII elongation, which is stalled at Ori-Lyt and PAN RNA promoter regions.

To gain insights into the gene regulation, we next examined occupancies of the LANA-interacting proteins by CUT&RUN in BCBL-1 (Fig. 4A) and BC-1 (Fig. S1). We selected proteins based on the availability of antibodies that have been proven for successful CUT&RUNs and immunofluorescence studies. The results showed that LANA-interacting proteins and enzymes (BRD4, ADNP, KMT2D, SMARCA5, and CHD4) are all colocalized with KSHV LANA on latent chromatin (Fig. 4A). As expected, the LANA-interacting proteins were highly enriched at the TR region when we compared with occupancies at the unique region (Table 1). The LANA sequence reads at TR were especially enriched at TR (Table 1). No comparable signals were seen with any other proteins tested or genomic regions. With the criteria based on relative enrichment at TR, we suggest that at least RNAPII, LANA, BRD4, and CHD4 localize at TR in latently infected cells. The lack of specific enriched peaks expected for transcription factor binding for the CTCF or SMC1 at TR also suggested no cohesin-mediated fixed genomic loops with TR. Noteworthy, occupancies of those enzymes at TR were not equal, even though those transcription factors were found to be in close proximity to LANA (Fig. 3A). The results indicated either highly heterogenic recruitment at an individual episomal level in an infected cell or dynamic protein recruitment at the individual TR copy within an episome. If the interacting proteins were equally recruited by LANA interactions, we expect peaks similar to LANA (Table 1).

**TABLE 1:** Sequence read enrichment at KSHV terminal repeat region^*a*^

Protein or histone modification	Fold enrichment at TR over the highest peaks at the unique region
Histone H3K27Ac	×12.6 (95/1,194)
Histone H3K27me3	×3.6 (56/203)
Histone H3K4me1	×0.5 (126/62)
Histone H3K4me2	×3.4 (225/778)
Histone H3K4me3	×12.2 (340/4,133)
KSHV LANA	×89.9 (1,423/127,975)
RNAPII (POLR2A)	×10.4 (388/4,036)
BRD4	×37.1 (122/4,530)
CHD4	×15.4 (55/496)
SMARCA5	×5.7 (105/597)
ADNP	×5.8 (203/1,174)
KMT2D	×5.8 (88/515)
CTCF	×2.0 (1,126/2,272)
SMC1	×5.8 (80/464)

^*a*^ Enrichment of sequence reads at TR over the unique region was calculated by dividing the highest sequence reads at TR by that of the unique region. The CPM normalized sequence read counts are also shown in parentheses.

### Nucleosome organization at origin of lytic DNA replication

The observation, in which LANA-interacting proteins (transcription factor complex for latent maintenance) occupied epigenetically active unique regions with poised RNAPII and the latent chromatins were not actively transcribed, suggested that pausing transcription initiation at the genomic region may have an important role for latency. Zooming the KSHV genomic region (22,000–30,000), encompassing Ori-Lyt and PAN RNA coding regions, where the KSHV latent chromatin possessed conserved active histone modifications among infected cells (Fig. 1B), showed that LANA, CHD4, ADNP, SMARCA5, and BRD4 are recruited next to the CTCF/SMC1 peak (Fig. 4B). The CTCF/SMC1 binding site also creates a small boundary of the transcription regulatory domain at the site (23) (Fig. 4B, CTCF binding sites marked with green shadow). The LANA complex occupied the H3K27Ac nucleosome localized next to the RNAPII peak (Fig. 4B, red shadow), suggesting that the H3K27Ac-nucleosome may play a critical role in stalling the RNAPII. Two K-Rta binding sites (37) were clearly positioned next to the LANA-bound nucleosome. Directionality and position of CTCF/SMC1 as well as previous Hi-C sequence reads (23) suggested that Ori-Lyt forms small genomic loops with the PAN RNA promoter region, and the genomic loop harbors the paused RNAPII (a combined figure with previous Hi-C studies is presented in Fig. S2). The RNAPII peaks at Ori-Lyt are the strongest, when we considered the TR copy number in all three PEL cell lines (Fig. 4B). The results suggested that the majority of infected episomes in a cell in PEL cell lines harbor the paused RNAPII at the same position (Fig. 4B; Fig. S1). Because K-Rta binding sites were positioned next to the LANA-bound H3K27Ac-modified nucleosome and the K-Rta protein complex contains SWI/SNF (Fig. 3), we next examined if K-Rta expression evicts the nucleosome and triggers transcription elongation. Indeed, the nucleosome occupancies were significantly decreased when we triggered the reactivation with K-Rta expression (Fig. 4C). Furthermore, the GRO-sequence showed strong induction of transcription elongation at the genomic region during reactivation (Fig. 2). Based on the conserved histone modifications (Fig. 1) and protein recruitments (Fig. 4; Fig. S1), local 3D genomic structure with the position of poised RNAPII (Fig. S2), and robust nascent RNA expression with reactivation stimuli (Fig. 2), we suggest that a KSHV latency–lytic switch is triggered by the initiation of transcription elongation at the Ori-Lyt through the recruitment of the K-Rta complex (Fig. 4D).

### Genomic interaction between TR and unique region

**Fig 5:**
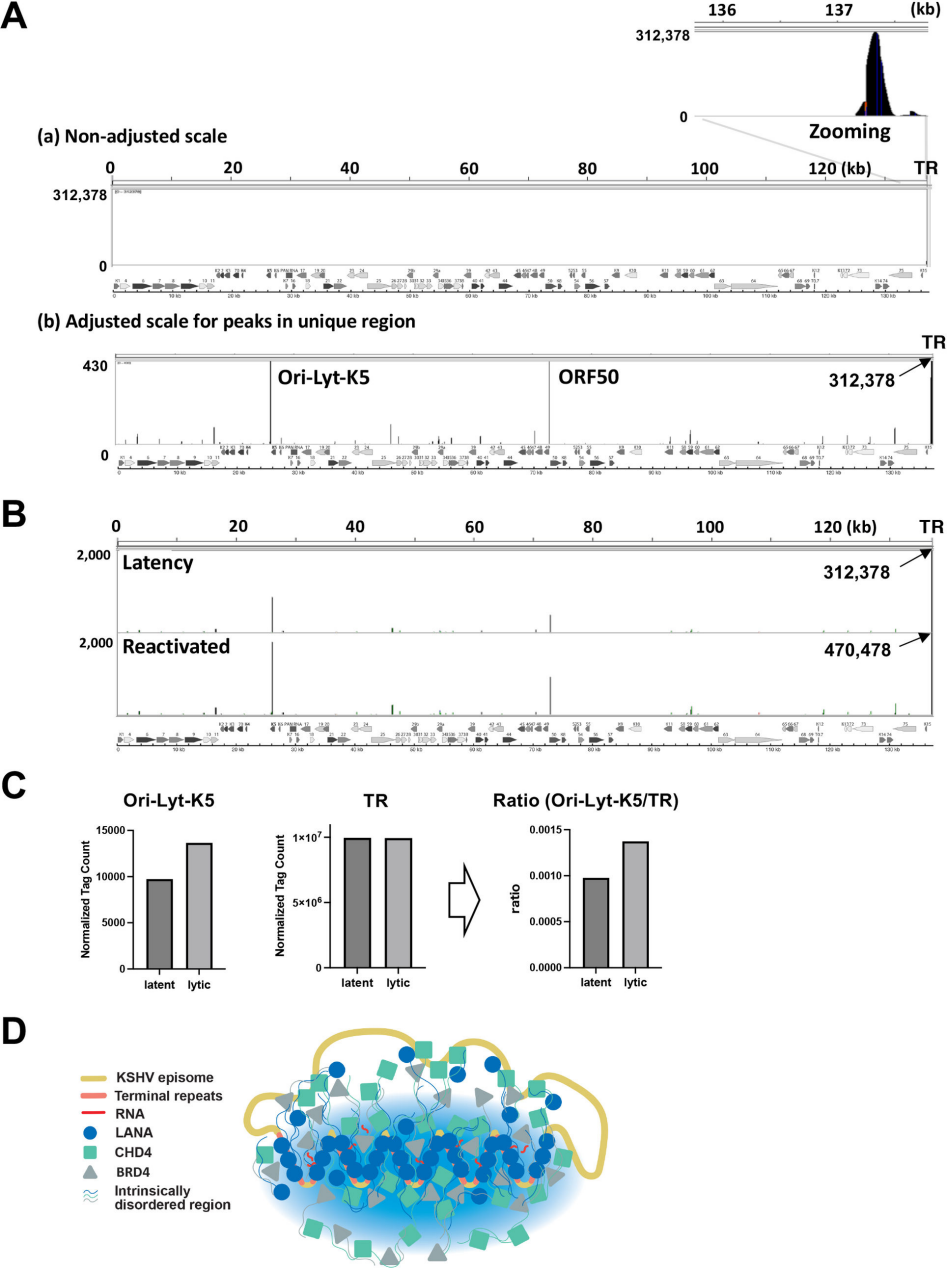
The terminal repeat does not form a fixed genomic loop with the unique region. (**A**) Position of TR ligated DNA fragments. The genomic looping with TR fragments was examined by isolating sequence reads from previous Hi-C data sets. The ligated DNA fragments with TR elements were aligned to the KSHV genome. The scale of the KSHV genome and ORF maps is shown at the bottom of the panel. A non-adjusted scale with a default setting, which is based on the highest peaks, and the adjusted scale to show peaks in the unique region (bottom panel) are presented. (**B**) Changes in looping frequencies by reactivation. Site and frequencies of ligated DNA fragments with TR are shown. The site of genomic loops was conserved, but frequencies of genomic looping with TR were increased. (**C**) Normalized tag counts found at the peak. The total number of tags was normalized to 10 million mapped tags. Peaks were identified by findPeaks, HOMER (v4.11). Normalized tag counts around the Ori-Lyt-K5 region (left) and TR region (center) are shown. The ratio of the normalized tag counts around the 26-kbp region to the TR region is also shown in the bar chart (right). (**D**) Summary of Fig. 4 and 5. A putative LANA/TR nuclear body function as a gene regulatory domain is presented. LANA is accumulated at the TR region with direct DNA bindings that increase the concentration of LANA (blue circle) and interacting proteins such as CHD4 (green square) and BRD4 (gray triangle). Locally concentrated transcription-related proteins facilitate the regulation of lytic gene promoters with LANA.

Having similar protein complexes recruited at selected genomic loci in the unique region and TR, we asked if these two genomic regions were located in particularly closer proximity in the 3D genomic structure. To study this, we reanalyzed previous Hi-C sequence data sets with TREx-K-Rta BCBL-1 cells (23). We first isolated KSHV TR fragments and mapped the position of ligated KSHV DNA fragments onto the unique region of the KSHV genome. The results showed that a significant majority of TR fragments (>99.5%) were ligated with other TR fragments (Fig. 5A), suggesting that individual TR units are highly compacted with each other. The result agreed with studies on LANA NBs with super-resolution fluorescence microscope analyses (38) and indicated that KSHV TR fragments formed a self-aggregate with LANA and LANA-interacting proteins. Even though overall frequencies are much less (<0.5% of total TR genomic loops), two genomic regions formed genomic loops more frequently with TR (Fig. 5A). These regions were within the Ori-PAN RNA transcription regulatory domain (Fig. S2) and near the K-Rta promoter region. The induction of KSHV reactivation by K-Rta expression increased the overall KSHV-KSHV genomic loops (39), which also increased the genomic interaction with TRs (Fig. 5B). To further quantitatively assess the interaction between TR and the unique region in latent and reactivated cells, we calculated the normalized read counts for each condition and evaluated the ratio of read counts for the unique regions to TR regions. The ratio increased in lytic-infected cells, suggesting an increasing interaction between TR and the unique region during reactivation (Fig. 5C).

Considering CTCF to be frequently localized at epigenetically active genomic regions, having no CTCF binding sites within 25 kb+ of highly active DNA fragments (Table 1) would be a unique genomic feature. This design should make the TR a mobile enhancer for KSHV inducible genes, because the TR fragments are maintained to be in proximity to the inducible viral promoters with the episomal genomic structure. In summary, TR is a flexible large protein storage containing LANA and its interacting proteins such as CHD4 and BRD4. The protein/TR aggregates were then maintained in proximity to inducible promoters, especially to the Ori-PAN RNA region and K-R promoter region in 3D (Fig. 5D).

### TR supports LANA and K-Rta transcription function

**Fig 6:**
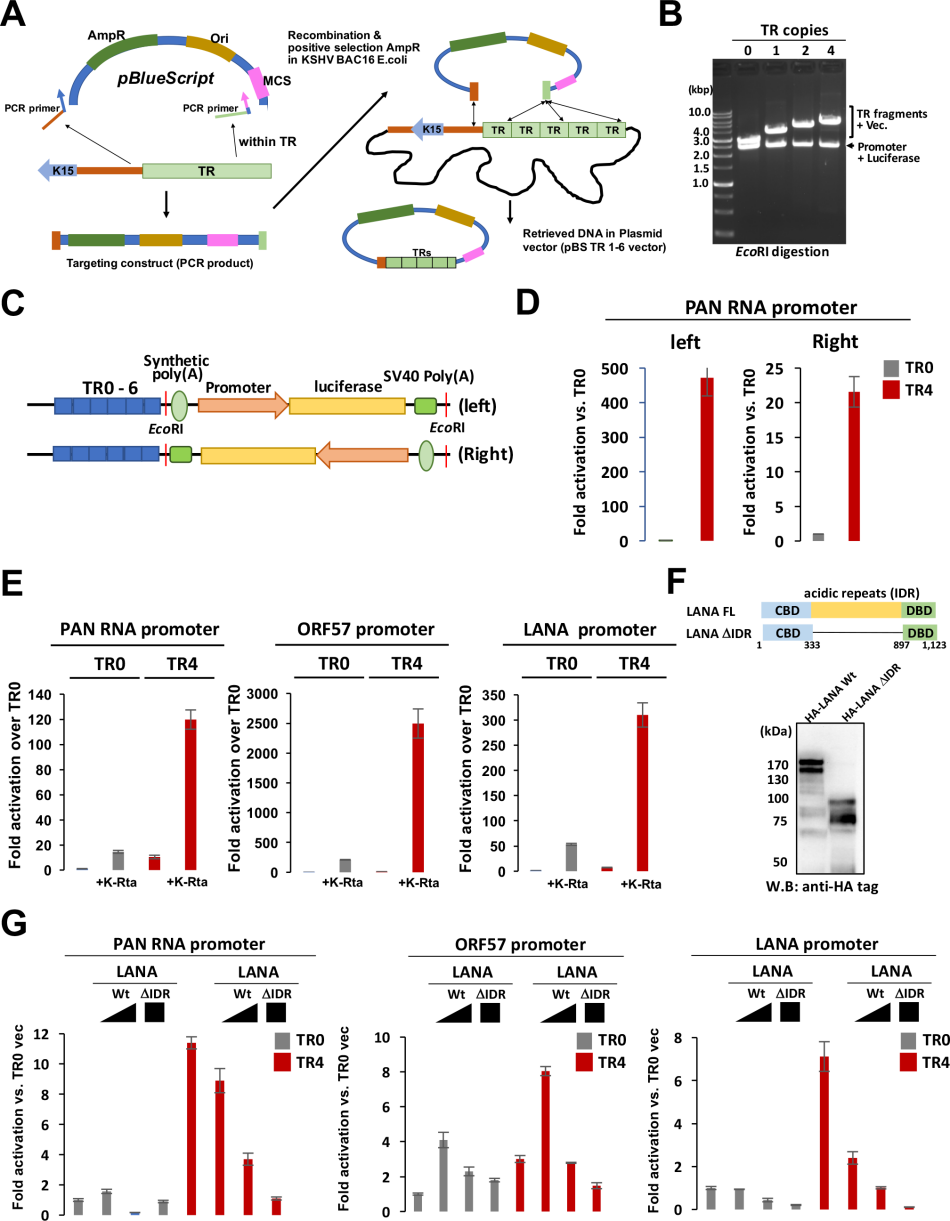
The terminal repeat fragment possesses an enhancer function. (**A**) Recombination mediated TR cloning. Schematic diagram of TR fragment cloning. Long primers with homology arms to the end of the unique sequence and TR sequence were used to amplify the pBlueScript plasmid. The amplified DNA fragment was transformed into BAC16 containing GS1783 for recombination. (**B**) Confirmation of TR insertion. The EtBr-stained agarose gel is shown. The copy number of TR was determined by an 801-bp incremental size increase. (**C**) Schematic diagram of the luciferase reporter construct. Luciferase reporters were amplified from the pGL3 vector and cloned into *EcoR*I sites in two orientations (left and right). (**D**) TR activates the PAN RNA promoter in an orientation-independent manner. Reporter constructs were transfected into 293FT cells, and luciferase activity was measured 48 h post-transfection. The luciferase value with TR0 was normalized as 1, and fold activation is shown. The standard deviation with triplicated samples is shown. (**E**) TR enhances the K-Rta transactivation function. Indicated reporter constructs (right direction) were cotransfected with K-Rta expression plasmid in 293FT cells. Fold activation over TR0 with vector control transfection is shown. Standard deviation with triplicated samples is shown as error bars. (**F**) Diagram and expression of LANA mutant. The LANA acidic domain with acidic repeat domain deletion was constructed for the reporter assay. The position of amino acid deletion and expression in 293FT were shown. (**G**) TR enhances LANA’s transcription function. Indicated reporter constructs (1 µg) were cotransfected with either full-length LANA expression plasmid (0.5 or 2.0 µg) or LANAΔIDR (2.0 µg). Luciferase values were measured 48 h post-transfection. For luciferase reporter assays, error bars with standard deviation for triplicated samples were shown. We performed reporter assays at least three times for each setting, and representing results of one of the replicates are shown. Standard deviation with triplicated samples is shown as error bars.

The H3K27Ac histone modification and enrichment of transcription enzymes suggest that TR may possess a transcription regulatory function when it localizes proximity to a promoter. To study this directly, we first cloned TR from KSHV BAC16 by homologous recombination with recombineering technique (40) (Fig. 6A). Homology arms are included in the long primer sequence (Table 2), and the primers were used to amplify the pBlueScript plasmid vector. The resulting PCR fragment containing plasmid DNA’s origin, ampicillin-resistant cassette, and partial multiple cloning sites was transduced into BAC16 containing *Escherichia coli*. The successful recombination generates circular plasmids in BAC containing *E. coli,* which outgrowths via high copy origins of plasmid DNA replication. The ampicillin-resistant colonies were then screened for the presence of TR, and the number of TR copies was examined by the size of plasmid DNA after restriction enzyme digestion (Fig. 6B). To study the association of increased TR copy numbers with transcription function, we isolated the pBlueScript-TR (pBS-TR) vector encoding 0, 2, 4, and 6 copies of TRs. We then cloned the luciferase reporter cassette into the pBS-TR vectors. KSHV reporter libraries (26) generated with the pGL3 basic vector were used as a PCR template. The reporter cassette includes synthetic poly(A) sites upstream of the inserted promoter and SV40 poly(A) sites downstream of the luciferase gene (Fig. 6C). We cloned reporter constructs in two orientations (left and right: Fig. 6C) and examined effects on directionality. The results demonstrated that the basal KSHV PAN RNA promoter activity was increased independent of the orientation in 293FT cells in the presence of the TR fragment. The results suggested that the TR region has an enhancer activity since enhancers are known to act independently of the orientation (41, 42) (Fig. 6D). However, the TR-left PAN promoter, in which TR fragments are physically closer to the PAN RNA promoter, showed higher luciferase activity. Including the TR fragment in the reporter also synergized K-Rta-mediated reporter activation (Fig. 6E). On the other hand, while the presence of TR increased basal promoter activity (TR0 vs TR4 with vector control in Fig. 6G), LANA acquired a stronger gene repression function only in the presence of TR. The effects were exaggerated when we deleted the LANA IDR domain (Fig. 6G). The results suggested that TR fragments increased the basal levels of transcription activity (TR0 vs TR4) and enhanced the K-Rta transactivation function synergistically. To study the association between TR copy number and transcription activity, we cotransfected reporter with TR2, 4, and 6 with K-Rta. The results showed that increasing the TR copy number enhanced promoter activity; however, the effects were not always linear in 293FT cells. The results showed that the enhancement of the promoter activity was saturated with four copies of TR in the reporter assay (Fig. S3).

**TABLE 2:** Primer and DNA fragments used in this study^*a*^

Name of primer	Sequence 5′ → 3′
Homology arm terminal repeats sense for retribution	GGGGACGCCGCCGGGGCCTGCGGCGCCTCCCGCCCGGGCATGGG GCCGCGCGCCGCCTCAGGGCCCGGCGCGGCCG**ACTAGTGGATCCCCCGGGCTGCAGGAATTCGATATC**
Homology arm terminal repeats antisense for retribution	CATTTCCAGGATACTCTTCATCTTATCTGTTTTTTGCAGCTGTAACAA TTTACGAGCCTTGTATCCGGAATATTTATG**CTGGGAAAACCCTGGCGTTACCCAACTTAATCG**
CUT&RUN qPCR	
Ori-RNA_S	ATACCCAGGTGGGTGAAC
Ori-RNA_AS	AATTATAGAATTGCAGCTGGGT
Control region (ORF25)-S	GGAGAGGTCAAGACGTGAAAC
Control region (ORF25)-AS	GGACGTGAAGGCTAGTCTAGTA
Reporter plasmid cloning	
pGL3 basic EcoRI S	AAA***gaattc***TTGGAGCGGCCGCAATAAAATATCTTTATTTTCATTAC
pGL3 basic EcoRI AS	AAA***gaattc***TCAAGGGCATCGGTCGACGGATCCTTATCG
pGL3 basic PstI S	AAA***ctgcag***TTGGAGCGGCCGCAATAAAATATCTTTATTTTCATTAC
pGL3 basic PstI AS	AAA***ctgcag***TCAAGGGCATCGGTCGACGGATCCTTATCG

^*a*^ Longer primers that anneal to the plasmid template are underlined with bold case. The restriction enzyme sites are shown in small italic case.

**Fig 7:**
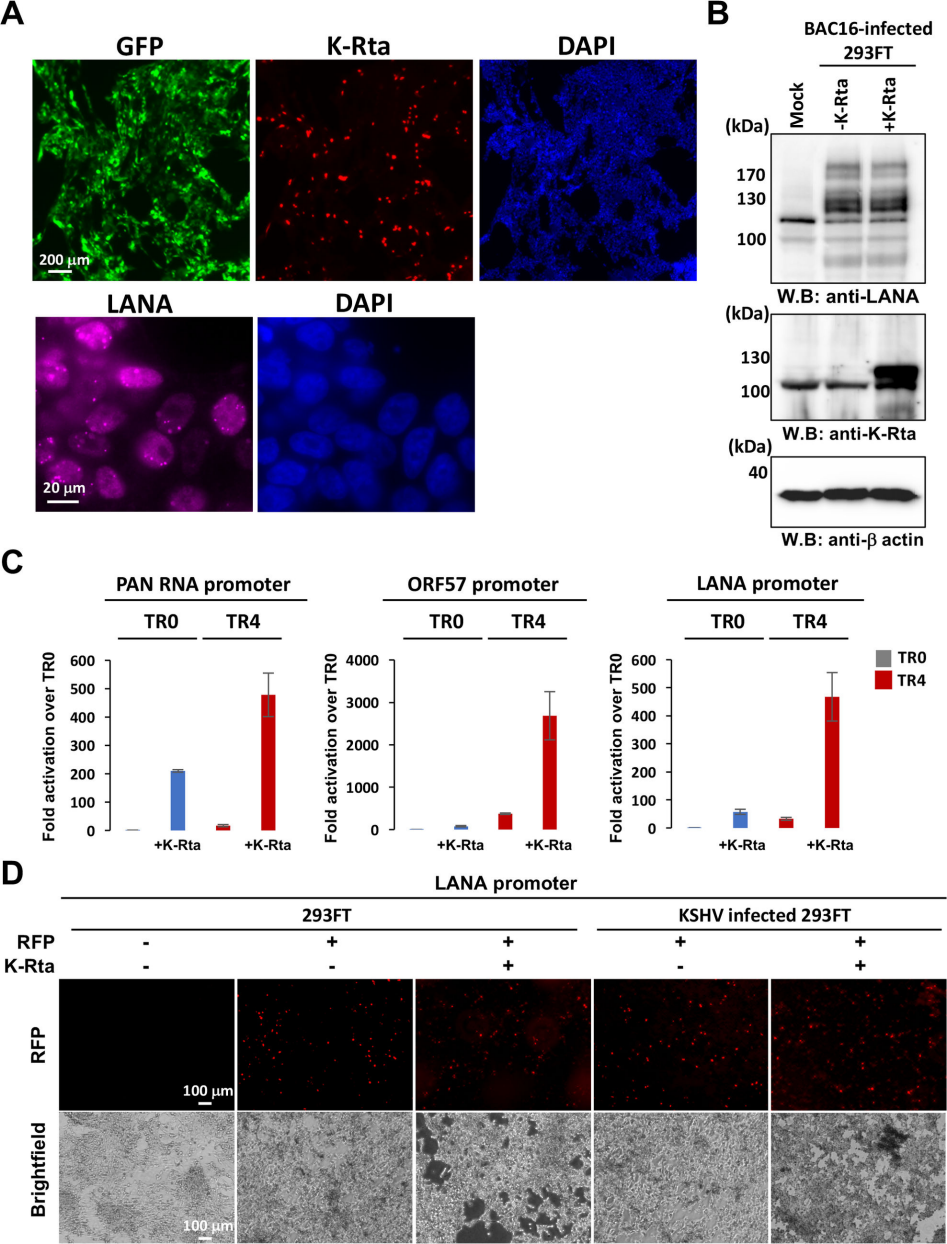
Terminal repeat fragment possesses enhancer function in KSHV-infected 293FT cells. (**A**) Generation of KSHV-infected cells. The BAC16-Wt KSHV was infected in 293FT cells and selected with hygromycin. The BAC16 stable 293FT cells were stained with the indicated antibody after transfection of the K-Rta expression plasmid. DNA was stained with 4′,6-diamidino-2-phenylindole. The scale is shown in the panel. (**B**) Immunoblotting. Infection and expression of K-Rta were confirmed by immunoblotting. Indicated antibodies were used for immunoblotting. β-Actin served as the loading control. The position of protein size markers is indicated at the left of the panels. (**C**) Luciferase assay. Indicated reporter constructs were transfected with either vector control or K-Rta expression plasmid. Luciferase values were measured 48 h after transfection. The luciferase unit with TR0 reporter with vector control was designated as 1, and fold activation is shown. Standard deviation with triplicated samples is shown as error bars. (**D**) Confirmation of transfection efficacies. Images of 293FT and KSHV-infected 293FT cells are shown. RFP-expressing plasmid was cotransfected with reporter and/or K-Rta expression plasmid, and transfection efficacies were examined by RFP signals. Images were captured 48 h after transfection. The scale bar is shown in the left panels.

Finally, the enhancer activity was tested with KSHV-infected 293FT cells. We first prepared infectious KSHV from BAC16-Wt iSLK cells and generated KSHV-infected 293FT cells with hygromycin selection (Fig. 7A). The KSHV infection was confirmed with immunofluorescence staining and immunoblotting (Fig. 7A and B). The KSHV-infected 293FT cells were then cotransfected with K-Rta expression plasmid with reporter constructs and examined enhancer activity. The results again showed that TR enhanced K-Rta-mediated transcription function synergistically, even more so than in non-KSHV-infected 293FT cells (Fig. 7C). The transfection efficacies were determined by the proportion of the RFP-positive cell population and showed that there were no obvious differences in transfection efficiency (Fig. 7D).

Taken together, we propose that KSHV TR is an enhancer for both transcription activation and repression with K-Rta and LANA, respectively. Our study suggests that KSHV evolved a very clever design of the KSHV enhancer, which encodes arrays of LANA DNA-binding sites to create phase-separate LANA NB at TR; this mechanism positions LANA to control KSHV lytic gene promoters during latency.

## DISCUSSION

While KSHV promoter activity has been measured in isolated reporter plasmids and a number of key cellular transcription factors were identified as important regulators for the KSHV latency–lytic switch (26, 43–46), a mechanism through which many viral promoters are synchronously regulated remains unclear. This study aimed to provide insight into the spatial and temporal KSHV gene regulation by focusing on the transcription function of TR. The TR region takes up to one-fifth of the highly crowded KSHV genome (38, 47).

To identify critical regulatory domains of latent chromatin, we first established a histone modification map with CUT&RUN analyses (48) (Fig. 1). Our CUT&RUN showed the enrichment of each transcription factor or histone modification as a relative peak across the KSHV genome. Because CUT&RUN produces a significantly lower background than ChIP-seq and is very sensitive to the specificity of antibodies, the peak height does not always indicate an abundance of the recruited protein at the specific sites. Differences in affinity and specificity among antibodies and also differences in the total amount of the protein in the cell make significant differences in releasing DNA fragments via incubation with protein-A/G-micrococcal nuclease (MNase). Accordingly, our focus on the CUT&RUNs was to identify protein recruitment sites on the KSHV genome comprehensively and compare the position of peaks among naturally infected cells.

The histone modification predicts which genomic regions are accessible to DNA-binding proteins. The CTCF, SMC1A, and Hi-C data also helped us to identify key genomic regions that formed a higher density of genomic hubs (23). Among active histone marks, H3K4me1, H3K4me3, and H3K27Ac are known to be present at enhancers (49, 50). Subsequent studies showed that H3K4me1 is a marker for poised enhancers. When the poised enhancer is activated, the enhancer begins to possess H3K27Ac marks with or without H3K4me1 (51). However, the concept of enhancers has been reconsidered in recent years, and recent models emphasize more for the presence of regulatory elements (52). With this definition, many regulatory elements with enhancer activity (the ability to enhance distal transcription) can also work as promoters, while gene promoters, defined by the location near the transcription start sites of the coding sequence, may also have enhancer activity (52–55). In this study, we described TR as an enhancer because we showed that the TR region served as a platform to recruit transcription regulatory proteins (Table 1) and serve as a regulatory domain for KSHV inducible genes (Fig. 6).

We initially expected highly conserved H3K27me3 marks on KSHV latent chromatins, since latent chromatin is largely silenced. Studies by others elegantly demonstrated the recruitment of the PRC1/2 complex on KSHV genomes in iSLK cells (56–58). To our surprise, we found that the degree and position of H3K27me3 marks were varied among naturally infected three PEL cell lines (Fig. 1B) and also experimentally infected iSLK cells. Compared with BC3 cells, the amount of H3K27me3 at the late gene cluster in BC1 was much less, and the position of the H3K27me3 also varied among cell lines. Considering that each PEL cell harbors 30+ episomes and studies have been performed with cell population, we think that only a selected fraction of infected episomes may possess H3K27me3 modifications, yet KSHV episomes are not actively transcribed in PEL cells. Accordingly, we would like to suggest that pausing RNAII elongation at the unique region but not global chromatin condensation(s) may be the major contributor to silencing inducible gene transcriptions. On the other hand, active histone modifications such as H3K27Ac and H3K4me3 were more conserved at specific genomic regions in PEL cell lines and experimentally infected cells. This suggests that maintaining a specific active genomic domain is the strategy that KSHV has evolved for maintaining inducible chromatin in the presence of K-Rta. Importantly, these active regions possess the major K-Rta and LANA binding sites, and the K-Rta DNA-binding sites are precisely positioned next to the LANA complex-tethered nucleosome at both Ori-Lyt regions. For that, a high LANA protein concentration at TR may help to ensure LANA and LANA-interacting protein recruitment at the LANA binding sites in the unique region for latency establishment and maintenance by distantly regulating the inducible promoter. Although more mechanistic studies have to be followed, the recruitment of SWI/SNF via K-Rta DNA binding is expected to evict the nucleosome, hence the LANA complex from the Ori-Lyt DNA (Fig. 4D). Indeed, an earlier study showed establishing nucleosome-free loci at K-Rta promoter regions during reactivation (59). Together, we propose that the eviction of LANA-tethered nucleosome at Ori-Lyt, where RNAPII paused, is a well-designed and highly efficient KSHV latency–lytic switch mechanism.

A combination of previous proteomics studies on LANA and K-Rta allowed us to draw the putative protein interaction maps (Fig. 3). We showed that cellular proteins that interact with LANA in latently infected cells may also accommodate enzymes that interact with RNAPII and K-Rta during reactivation. Multiple copies of TR recruited a significant amount of LANA and BRD4. Those two proteins have IDR domains and can attract many other histone enzymes as protein hubs. We speculate that large and highly disordered acidic repeat regions (IDR) of LANA play an important role in capturing such enzymes at TR. KSHV LANA (60) and BRD4 (11) are known to form phase-separate protein condensates that should further facilitate TR fragments to be compartmentalized, which is indeed seen in Hi-C (Fig. 5). The mechanism is analogous to a system operated at cellular super-enhancer with the interaction between two IDR-containing proteins, BRD4 and MED1 (11). Importantly, KSHV K-Rta interacts with MED1 during reactivation (Fig. 3), and KSHV LANA associates with BRD4 at TR and Ori-Lyt region (Fig. 4; Table 1). Thus, TR is a well-poised enhancer for KSHV lytic gene promoters, and viral gene promoters are activated by the recruitment of MED1, a component of the mediator complex, by K-Rta recruitment in the unique region. The recruitment of MED1 expects to create many inducible genomic loops among BRD4-bound regions [TRs and RNAPII poised regions in the unique region (Fig. 4; Fig. S1)]. Importantly, we demonstrated such a genomic looping shift during reactivation, in which the KSHV 3D genomic structure was more compacted by the generation of larger transcription regulatory domains, and 3D genomic structures became squeezed doughnut shapes (23).

Suppose the activity of KSHV TR is essential for lytic gene expression. How is the KSHV TR initially activated during *de novo* infection and maintained to be activated in the infected host cells? Does constitutively active NF-κB and STAT pathways frequently seen in KSHV-infected cells contribute to active episome maintenance? We speculate that large LANA NBs are like a “solar panel” to extract energy by intercepting cellular signaling events via a highly concentrated LANA/BRD4 complex at TR. The highly concentrated IDR proteins would absorb transcription enzymes from nearing cellular transcription factors because the affinity of interaction among cellular transcription factors and enzymes (i.e., SWI/SNF) is relatively weak in order to share the coactivators among other cellular transcription factors for the dose-dependent regulation, except viral transcription factors like K-Rta (61). The combination of multiple copies of TR fragments and a large unstructured acidic repeat in LANA seems to be designed to concentrate the protein complex near the KSHV unique protein-coding regions. Consistent with this, we have seen a number of transcription factors (e.g., STAT3, p65, IRF4, and BATF) and coactivator enzymes (SMARCA4, SMARCC1) and other proteins that form dynamic and flexible protein complexes colocalize with the LANA NBs in BCBL-1. Similar observations have also been reported (62–66). We also showed that inhibition of STAT3 activation at the beginning of KSHV *de novo* infection attenuated KSHV reactivation (67). While these ideas await further examination, LANA/TRs may, in return, function like a sponge to weaken the ability of cellular TFs to directly stimulate KSHV reactivation, which should help to maintain the latent state for immune evasion. Related to this, we recently showed that the rapid LANA degradation exposed KSHV episomes to the cGAS DNA sensor and consequently triggered KSHV episome degradation (68).

In summary, we suggest that KSHV TR is an important regulatory domain for KSHV inducible genes by enhancing the transcription function of the KSHV proteins. In contrast to cellular enhancers that are bound by multiple transcription factors, perhaps the KSHV enhancer is predominantly regulated by the LANA NB via a clever genomic design. The regulation of dynamic protein interaction at Ori-Lyt during reactivation and revealing how KSHV episome maintains an epigenetically active state with TR should provide an important target to intercept KSHV replication cycles.

## MATERIALS AND METHODS

### Chemicals, reagents, and antibodies

Dulbecco’s modified minimal essential medium (DMEM), fetal bovine serum (FBS), phosphate-buffered saline (PBS), Trypsin-EDTA solution, and 100× penicillin–streptomycin–L-glutamine solution were purchased from Thermo Fisher (Waltham, MA). Puromycin and G418 solution were obtained from InvivoGen (San Diego, CA). Hygromycin B solution was purchased from Enzo Life Science (Farmingdale, NY). The following antibodies were used for CUT&RUN, immunoblotting, and flow cytometry: rabbit anti-BRD4 (Cell Signaling, E2A7X), rabbit anti-RNAPII (Millipore, clone CTD4H8), rabbit anti-H3K27ac (CST, clone D5E4), rabbit anti-H3K4me3 (Cell Signaling, clone C42D8), mouse anti-β-actin (Santa Cruz, 47778), rabbit anti-gp130 (CST), and rabbit IgG (CST, clone DA1E).

### Cell culture

293T cells were grown in DMEM containing 10% FBS and 1× Pen-Strep-L-Gln at 37°C with air containing 5% carbon dioxide. BAC16-Wt stable iSLK cells were maintained in DMEM containing 10% FBS, 400 µg/mL hygromycin B, 250 µg/mL G418, and 1× Pen-Strep-L-Gln at 37°C with air containing 5% carbon dioxide. The KSHV BAC16-Wt infected 293FT cells were established after infection for BAC16-Wt virus with hygromycin selection (1,000 µg/mL) for 2 weeks.

### Preparation of pTR-reporter construct

KSHV TR fragments were cloned by adapting the recombineering technique (40). Homology arms with one targeting the unique region and another one targeting the TR region were designed and used to amplify the pBlueScript plasmid fragment. The linear PCR fragment that encodes a portion of multiple cloning sites, the origin of plasmid replication, and ampicillin-resistant cassette was purified from DNA agarose gel. Purified DNA fragments were then introduced to BAC16 containing GS1783 *E. coli* for red recombination (69). The ampicillin-resistant colonies were isolated and cultured in 32°C overnight. Outgrowing plasmids were purified with Qiagen Mini-prep kits. Purified plasmids were used for restriction enzyme digestions to identify the number of TR copies cloned. The pBluescript plasmid containing TR2, 4, or 6 was then used to transform into stbl3 strain (Invitrogen) for further plasmid amplification.

Luciferase reporter fragments were amplified from the pGL3 KSHV promoter library with primers listed in Table 1. The amplified reporter DNA fragment was cloned into pBS-TR multiple cloning sites (*Eco*RI, *Pst*I, or *Hinc*II). The luciferase reporter fragments were cloned for two different orientations. The schematic diagram was also presented in a figure. The expression plasmid for pcDNA HA-K-Rta and pcDNA HA-LANA was described previously (70, 71). The position of cloned KSHV promoter fragments is listed previously (26). For the generation of HA-LANA IDR deletion expression plasmid (pcDNA HA-LANAΔIDR), the DNA fragment was synthesized (IDTDNA) and cloned into the pcDNA HA-vector. Synthesized DNA fragments are listed in Table 1.

### Cleavage under targets and release using nuclease

CUT&RUN (48) was performed essentially by following the online protocol developed by Dr. Henikoff’s lab with a few modifications. Cells were washed with PBS and wash buffer [20 mM HEPES-KOH pH 7.5, 150 mM NaCl, 0.5 mM spermidine (Sigma, S2626), and proteinase inhibitor (Roche)]. After removing the wash buffer, cells were captured on magnetic concanavalin A beads (Polysciences, Pennsylvania, USA) in the presence of CaCl_2_. Beads/cell complexes were washed three times with digitonin wash buffer (0.02% digitonin, 20 mM HEPES-KOH pH 7.5, 150 mM NaCl, 0.5 mM spermidine, and 1× proteinase inhibitor), aliquoted, and incubated with specific antibodies (1:50) in 250 µL volume at 4°C overnight. After incubation, the unbound antibody was removed with digitonin wash buffer three times. Beads were then incubated with recombinant protein-A/G-micrococcal nuclease (pAG-MNase), which was purified from *E. coli* in 250-µL digitonin wash buffer at 0.5  µg/mL final concentration for 1 h at 4°C with rotation. Unbound pAG-MNase was removed by washing with digitonin wash buffer three times. Pre-chilled digitonin wash buffer containing 2 mM CaCl_2_ (200 µL) was added to the beads and incubated on ice for 30 min. The pAG-MNase digestion was halted by the addition of 200  µL 2× STOP solution (340 mM NaCl, 20 mM EDTA, 4 mM EGTA, 50 µg/mL RNase A, and 50 µg/mL glycogen). The beads were incubated with shaking at 37°C for 10 min in a tube shaker at 300 rpm to release digested DNA fragments from the insoluble nuclear chromatin. The supernatant was then collected by removing the magnetic beads. DNA in the supernatant was purified using the NucleoSpin Gel & PCR kit (Takara Bio, Kusatsu, Shiga, Japan). Sequencing libraries were prepared from 3 ng DNA with the Kapa HyperPrep Kit (Roche) according to the manufacturer’s standard protocol. Libraries were multiplex-sequenced (2  ×  150 bp, paired-end) on an Illumina NovaSeq 6000 system to yield ~15 million mapped reads per sample.

CUT&RUN sequence reads were processed with fastp (72) and aligned to the human GRCh38/hg38 and KSHV reference genome (NC_009333.1) with Bowtie2 and yielding mapped reads in BAM files (73). We applied CPM normalization to show chromatin modification in BCBL-1 in Fig. 1A. Both raw sequenced data and CPM normalized data sets are available under accession number GSE241949. We also confirmed the identical shape of peaks with a spike-in normalization with *E. coli* DNA genomic DNA from pAG-MNase incubation (48). Processed files with the spike-in normalization are also available upon request.

For CUT&RUN quantitative PCR (qPCR) studies, TREx-K-Rta BCBL-1 cells were induced for lytic gene expression by incubat)] to ling with doxycycline (1 µg/mL) and TPA (20 ng/mL) for 48 h. TREx-BCBL-1 cells induced or non-induced were used for CUT&RUN, and the amount of released DNA fragments was examined by qPCR with primers listed in Table 1.

FASTQ files for the Hi-C experiments were processed through the HiCUP (v0.7.4) pipeline (74) using the KSHV reference genome (NC_009333.1). In the pipeline, Bowtie 2 (version 2.3.5.1) was used to map valid Hi-C ditags with a single restriction fragment, allowing only unique high-quality alignments across the genome. Invalid Hi-C ditags, such as dangling ends and PCR duplicates, were removed by the HiCUP Filter script. Valid Hi-C ditags were aligned to the TR region of the KSHV genome with Bowtie2 (73) with al-conc option setting. Each forward and reverse read was independently mapped to the KSHV genome. The extracted paired reads were mapped again to the KSHV reference genome to identify three-dimensional genomic interaction with TR. The mapped reads were visualized with Integrative Genomics Viewer (75).

### Reverse transcription quantitative PCR (RT-qPCR)

Total RNA was extracted using the Quick-RNA miniprep kit (Zymo Research, Irvine, CA, USA). A total of 1 µg of RNA was incubated with DNase I for 15 min and reverse-transcribed with the High-Capacity cDNA Reverse Transcription Kit (Thermo Fisher, Waltham, MA, USA). The resulting cDNA was used for qPCR. SYBR Green Universal master mix (Bio-Rad) was used for qPCR according to the manufacturer’s instructions. Each sample was normalized to 18S ribosomal RNA, and the ddCt fold change method was used to calculate relative quantification. All reactions were ran in triplicate. Primer sequences used for RT-qPCR are provided in Table S1.

### STRING protein interaction visualization

Names of previously identified (A) LANA neighboring proteins with proximity biotin labeling (24) and K-Rta interacting complex (61), which is recruited to RNAPII during reactivation, were collected and visualized with STRING, a public database of known and predicted protein–protein interactions (33). The putative protein interaction among K-Rta and LANA protein complex during reactivation was also visualized with STRING after manually combining protein names together. The list of proteins based on previous analyses (*P* < 0.05) with chromatin regulatory function is all included in this analysis.

### Luciferase assay

HEK293FT or KSHVr2.19-infected HEK293FT cells were seeded onto 12-well plates at 2.0 × 10^5^/well. The cells were transfected with an expression plasmid encoding the HA-epitope tagged K-Rta, LANA, or together, along with pBS-TR reporter constructs. The TR reporter constructs encode a varied number of TR repeats and KSHV gene promoter, which is cloned in front of the luciferase coding sequence. Cell lysates were prepared with 1% TritonX-100 and 0.5% NP-40 in PBS 48 h after transfection. Luciferase activity was measured according to the manufacturer’s protocol by using Varioskan LUX (Thermo Scientific). At least three independent measurements were performed for each setting.

### Immunoblotting

Protein lysates from luciferase assays were subjected to 8% SDS-PAGE gel and transferred to PVDF membranes (Millipore-Sigma, St. Louis, MO, USA). Membranes were incubated with 5% non-fat milk at room temperature for 15 min for blocking and then incubated with the primary antibody at 4°C overnight. After washing the membrane with TBST three times, the membrane was then incubated with horseradish peroxidase-conjugated secondary antibody (Santa Cruz) at RT for 1 h. The final dilution of the primary antibody was 1:2,000 for anti-HA tag (Covance) and 1:3,000 for anti-β-actin (Millipore-Sigma) and K-Rta (76).

### Statistical analysis

Experimental replicates of at least three for each sample, including negative controls, were prepared whenever applicable. Results are shown as the mean ± SD from at least three independent experiments. Statistical analyses were performed using GraphPad Prism 9.4.1 software. A value of *P* < 0.05 was considered statistically significant.

## Data Availability

The CUT and RUN data were deposited in the NCBI Gene Expression Omnibus (GEO) database under accession number GSE241949.

## SUPPLEMENTAL MATERIAL

Supplemental figures jvi.01386-23-s0001.pdf Figures S1 to S3.

ASM does not own the copyrights to Supplemental Material that may be linked to, or accessed through, an article. The authors have granted ASM a non-exclusive, world-wide license to publish the Supplemental Material files. Please contact the corresponding author directly for reuse.
