# Supplementary material for: Kaposi’s sarcoma-associated herpesvirus terminal repeat regulates inducible lytic gene promoters
Source: J Virol. 2024 Jan 19;98(2):e01386-23. doi: 10.1128/jvi.01386-23 (PMC10878276; doi:10.1128/jvi.01386-23)
Supplement: Supplemental figures — Figures S1 to S3. [file jvi.01386-23-s0001.pdf]

BC-1

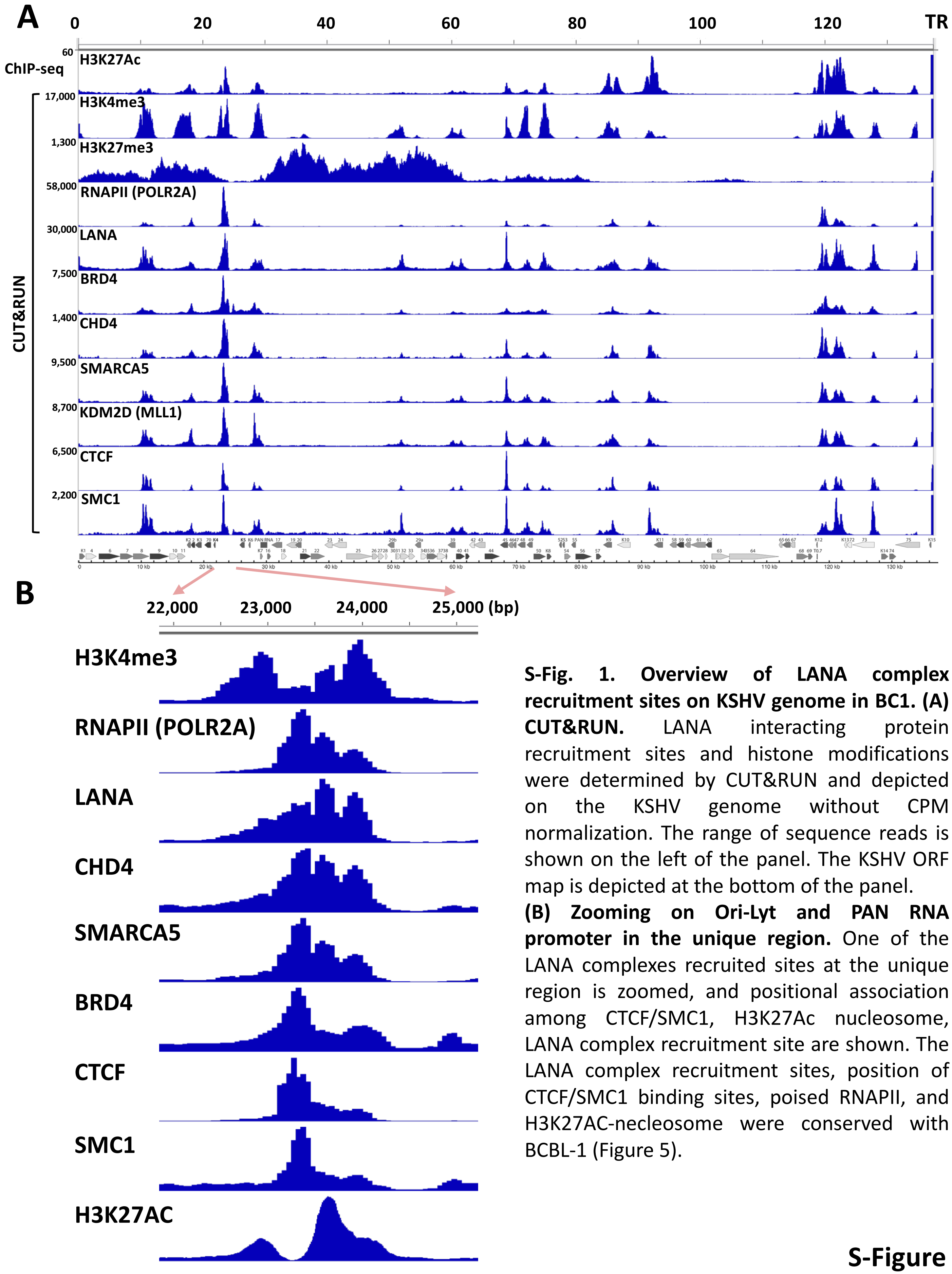

S-Fig. 1. Overview of LANA complex recruitment sites on KSHV genome in BC1. (A) CUT&RUN. LANA interacting protein recruitment sites and histone modifications were determined by CUT&RUN and depicted on the KSHV genome without CPM normalization. The range of sequence reads is shown on the left of the panel. The KSHV ORF map is depicted at the bottom of the panel. **(B) Zooming on Ori-Lyt and PAN RNA promoter in the unique region.** One of the LANA complexes recruited sites at the unique region is zoomed, and positional association among CTCF/SMC1, H3K27Ac nucleosome, LANA complex recruitment site are shown. The LANA complex recruitment sites, position of CTCF/SMC1 binding sites, poised RNAPII, and H3K27AC-necleosome were conserved with BCBL-1 (Figure 5).

S-Figure 1

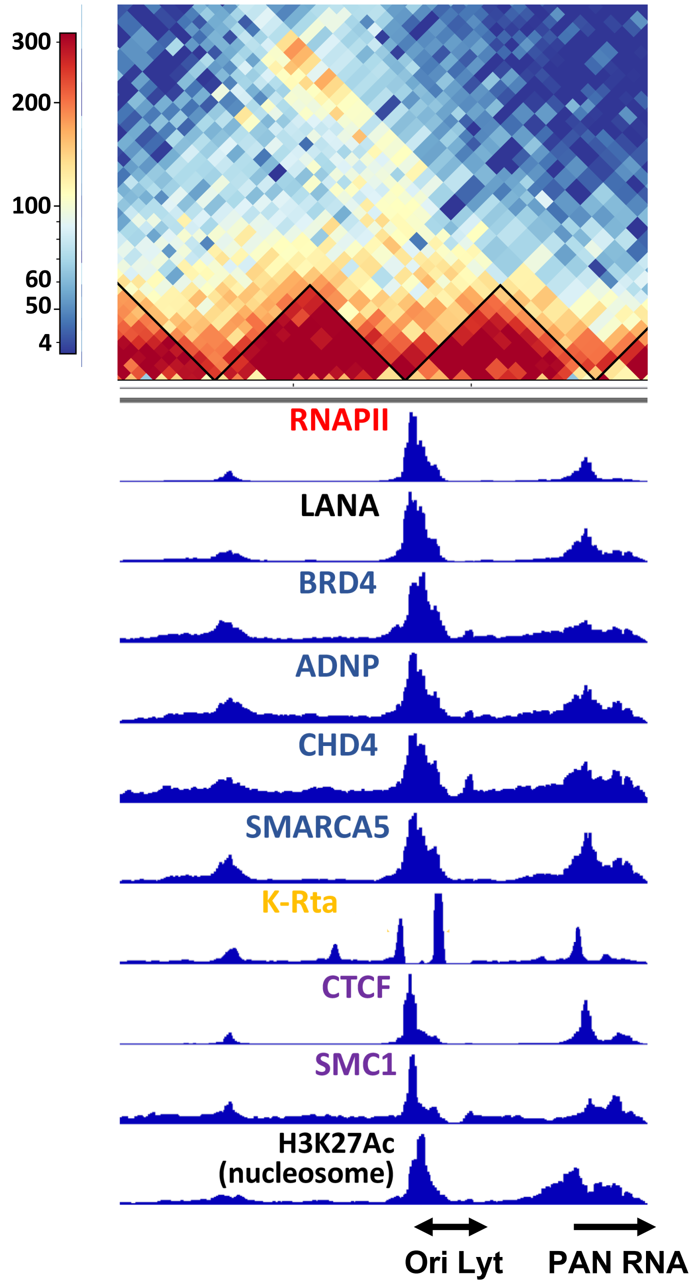

S-Fig. 2. Transcription regulatory domain at Ori-Lyt to PAN RNA region. Association of paused RNA polymerase II location and local genomic looping is shown by combining Hi-C data with CUT&RUN data sets. Heat map demonstrates frequencies of DNA ligation identified in Capture Hi-C in TREx-BCBL-1. Two other PEL cell lines, BC1 and BC3 have very similar transcription regulatory domain formations. Ori-Lyt region forms transcriptional regulatory domain with PAN RNA promoter and genomic domain is insulated by CTCF bindings. Note: Capture Hi-C heat map is prepared by rearranging previously published data sets (Campbell et al., JVI 2022).

S-Figure 2

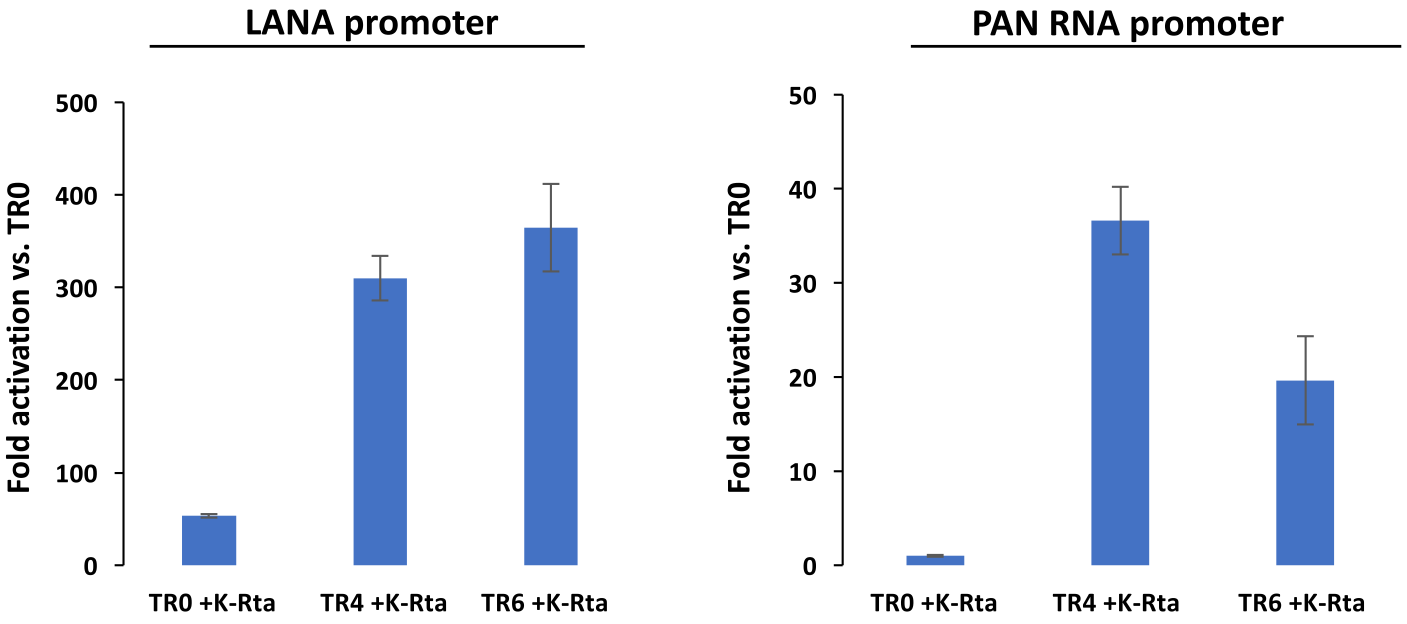

Luciferase reporter assay. Increased number of TR reporter was co-transfected with K-Rta expression plasmid, and examined association between TR copy and enhancer potency. Fold activation over no TR containing reporter plasmid with vector control is shown. Luciferase value with TR0 reporter with vector control was normalized as 1.

S-Figure 3
